# Neuroprotective Effects of Red Ginseng Saponins in Scopolamine-Treated Rats and Activity Screening Based on Pharmacokinetics

**DOI:** 10.3390/molecules24112136

**Published:** 2019-06-06

**Authors:** Jianbo Chen, Meijia Li, Di Qu, Yinshi Sun

**Affiliations:** Institute of Special Wild Economic Animals and Plants, Chinese Academy of Agriculture Sciences, Changchun 130112, China; chenjianbo00882@126.com (J.C.); limeijia_jiajia@163.com (M.L.); 15143162826@163.com (D.Q.)

**Keywords:** scopolamine, pharmacokinetics, red ginseng saponins, neuroprotective, ginsenosides

## Abstract

Ginseng has been used to alleviate age-related dementia and memory deterioration for thousands of years. This study investigated the protective effect of red ginseng saponins against scopolamine-induced cerebral injury. Meanwhile, pharmacokinetics of ginsenosides in normal and scopolamine-treated rats were compared. After scopolamine injection, glutathione, catalase and superoxide dismutase levels were significantly decreased when compared with control group. Compared with SA group, pretreatment of rats with red ginseng saponins could increase glutathione, catalase and superoxide dismutase level. Treatment with red ginseng saponins significantly decreased malondialdehyde level. In the pharmacokinetic analysis, a pattern recognition analysis method was used to investigate the pharmacokinetics of the absorbed compounds in blood. The pharmacokinetic parameters of Rg1, Rg2, Rh3, Rg5 and Rk1 in model group had higher area under the curve (AUC), mean residence time (MRT) and peak plasma concentration (Cmax) values; area under the curve (AUC) values and peak plasma concentration (Cmax) of model group was significantly different from that of normal group (*p* < 0.05). The Cmax value of Rk3, Rh1, Rh2 and Rh4 in model group was higher than normal group, but their AUC values were not significantly different. There was no significantly difference in time at Cmax (Tmax), AUC and Cmax values of Rb1, Rb2 Re, Rc, Rd and Rf between the model and normal group. 16 ginsenosides were grouped into three separate clusters according to principal component analysis (PCA) score plot based on pharmacokinetic data. The results suggested red ginseng saponins have significant protective effect against scopolamine-induced memory deficit and scopolamine-induced rats could lead to the changes of pharmacokinetic behaviors of ginsenosides.

## 1. Introduction

Alzheimer’s disease (AD) is a brain degenerative neurological disease, which is featured by memory decline and behavioral confusion [1,2]. According to relevant research, sustained oligemia or ischemic injury in the brain will lead to damage of cognitive function, memory and behavior [3,4]. Scopolamine (SA) was used extensively to screen for potential anti-dementia drugs because it can impair learning and memory [5,6]. According to some in vivo studies, AD and scopolamine treated subjects show an accumulation of reactive oxygen species (ROS) damage or reactive nitrogen species (RNS) damage (the two can be considered biomarkers of oxidative damage and nitrosative damage, respectively). The in vivo lipid peroxidation can be characterized by increased levels of malondialdehyde (MDA). It has been reported that glutathione (GSH) depletion could affect mitochondrial function. Therefore, brain oxidative stress can be recognized by enhanced expression/activity of the endogenous antioxidant enzymes such as superoxide dismutase (SOD), catalase (CAT) and GSH. Therefore, the addition of antioxidants may delay the development of AD and attenuate neural cell death induced by oxidative stress.

As a traditional Chinese medicine, panax ginseng has important pharmacological effects, which has been used in treating age-related dementia and memory deterioration for thousands of years [7,8,9]. The most common commercial ginseng is white ginseng (WG) and red ginseng (RG) [9]. RG has been increasingly used in traditional Chinese medicine prescriptions for different purposes due to its “warming effect”. RG is produced by steaming the non-peeled fresh roots and rhizomes of panax ginseng at 95–100 ℃ for 2–3 h and then drying [10,11]. This steaming process results in variations in the chemical constituents, especially for ginsenosides such as Rg5, Rg3, Rh2 and Rk1 (these ginsennosides only exist in RG rather than WG). Ginseng saponins have been demonstrated to have a wide range of pharmacological properties, such as anti-inflammatory, anti-aging, anti-fatigue and anti-tumor activities. Besides, anti-oxidant activity also has been reported [12,13,14,15,16,17]. Many studies have revealed that RG possesses better pharmacological effects than WG [18,19]. Because RG contains abundant less polar ginsenosides (such as Rg3, Rg5, Rh1, Rk1 and Rh4), that possess stronger biological activity potential than their parent compounds in WG [20,21,22]. Pharmacokinetic (PK) research has been conducted to account for the efficacy and toxicity of drugs. Currently, most PK studies of ginsennosides are conducted based on normal animal model or healthy human model, without the consideration of the pathological condition. However, it should be noted that the enzyme activity and membrane transporter capacity under pathological condition are significantly different from that under normal condition. Therefore, the pharmacokinetic behaviors of drugs under pathological condition may be changed [23,24,25]. Therefore, we examined the effect of red ginseng saponins (RGS) on oxidative stress status in the brain of scopolamine-treated rats. Meanwhile, we investigated the pharmacokinetics of the main ginsenosides in scopolamine-treated rats after oral administration of red ginseng saponins and we conducted a preliminary activity screening based on pharmacokinetics by pattern recognition approach, which is expected to provide reference for therapeutic effect evaluation.

## 2. Results and Discussion

### 2.1. Oxidation Stress Assessments

**Figure 1:**
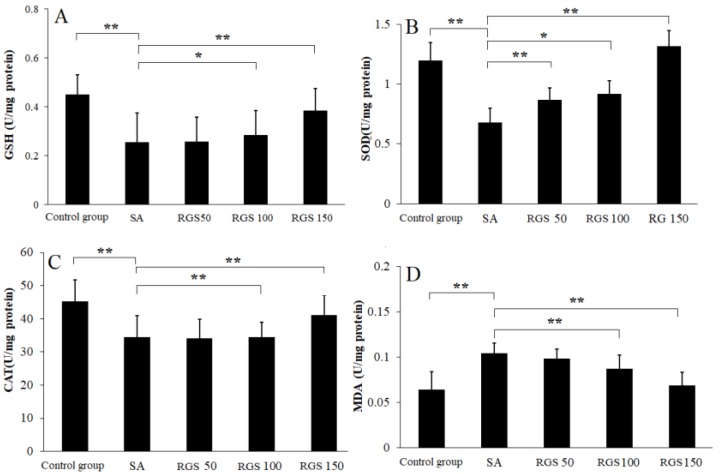
(**A**) glutathione (GSH), (**B**) superoxide dismutase (SOD), (**C**) catalase (CAT) and (**D**) malondialdehyde (MDA) levels in scopolamine-treated rats after pretreatment of red ginseng saponins (RGS). Values are expressed as mean ± SD (n = 6). * *p* < 0.05, ** *p* < 0.01. (one-way ANOVA followed by Tukey post hoc multiple comparison test).

After SA injection, the GSH level was significantly decreased when compared with control group. Compared with control group, the GSH level (0.45 ± 0.11 U/mg protein) was reduced to 0.25 ± 0.12 U/mg protein (*p* < 0.01) by SA injection. Compared with SA group, pretreatment of rats with RGS (50, 100 and 150 mg/kg) increased GSH levels by 1.01, 1.12 and 1.62 folds, respectively (Figure 1A). Similarly, compared with control group, SOD (1.23 ± 0.15 U/mg protein) and CAT (48.23 ± 6.52 U/mg protein) levels were decreased to 0.68 ± 0.12 and 34.52 ± 6.41 U/mg protein (*p* < 0.01) by SA injection, respectively. The decrease in SOD and CAT activity was reversed by the pretreatment of rats with RGS (50, 100 and 150 mg/kg) (Figure 1B,C). RGS with doses of 50, 100 and 150 mg/kg were observed with increased SOD levels of 0.87 ± 0.11, 0.92 ± 0.16 and 1.32 ± 0.13 U/mg protein, respectively. RGS with doses of 100 and 150 mg/kg were observed with increased CAT levels of 38.92 ± 4.61 and 42.73 ± 5.91 U/mg protein. SA administration significantly increased MDA level from 0.065 ± 0.02 to 0.15 ± 0.011 (U/mg protein) (*p* < 0.01) in comparison with control group. However, treatment with RGS (100 and 150 mg/kg) decreased MDA levels (0.089 ± 0.03 and 0.077 ± 0.02 U/mg protein) (*p* < 0.01) (Figure 1 D).

### 2.2. PK Study

#### 2.2.1. Validation of UPLC-MS/MS Method

**Figure 2:**
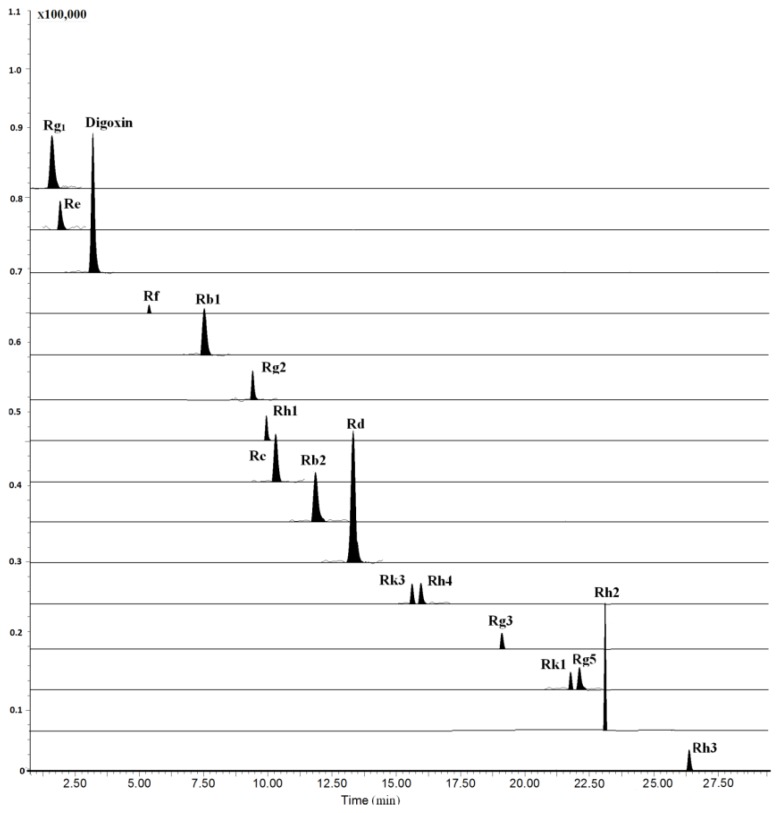
Typical multiple reactions monitoring (MRM) chromatograms of rat plasma collected at 4 h after oral administration of 100 mg/kg RGS.

The LC–MS/MS method was fully validated by assessing its LLOQ, linearity, precision, accuracy, recovery, and stability. All results were showed in Tables S1–S4. All analytes and internal standard (I.S.) were scanned by ESI^+^ and ESI^−^ mode, and the results showed that the relative intensity in ESI^−^ mode was significantly higher than that in ESI^+^ mode. Moreover, we investigated the composition of mobile phase for improving analyte ionization. It can be found that most of the analytes had abundant deprotonated molecular ions except for ginsenosides Rh3, Rh4 and Rk3 when the mobile phase was consisting of water and acetonitrile. Due to the poor ionization of Rh3, Rh4 and Rk3 for containing two double bonds at C-17 side-chain, the mobile phase additive of ammonium acetate and formic acid at different concentrations were investigated, higher sensitivity and ion intensity of the three ginsenosides were observed, wherein the 0.1 mM ammonium acetate solution served as the optimal mobile phase, while [M+CH3COOH-H]^−^ was selected as their precursor ions of MRM transitions (Figure 2). The optimized mass conditions are shown in Table 1.

#### 2.2.2. Effect of SA on PK of Different Ginsenosides

**Figure 3:**
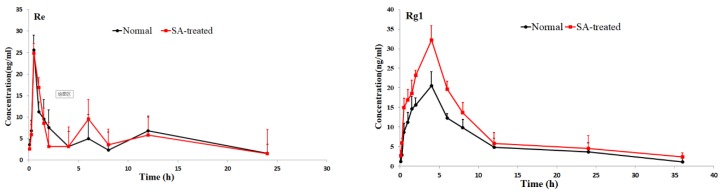

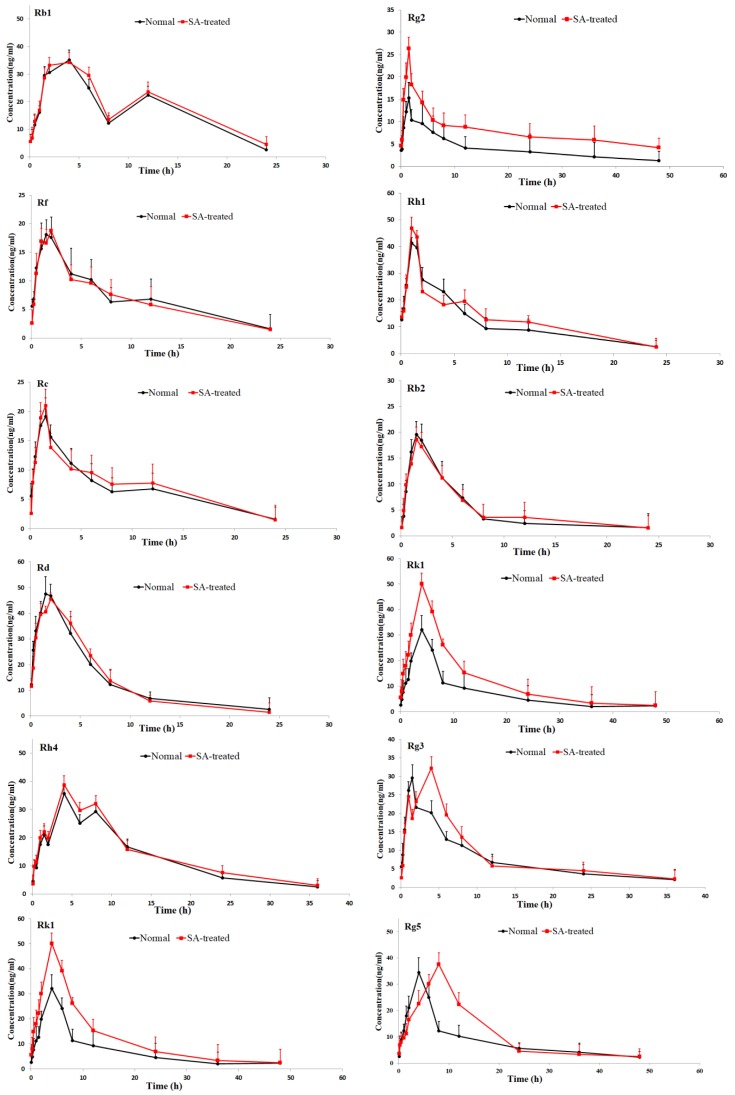

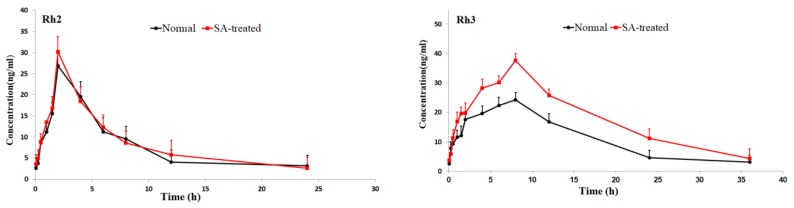
Mean plasma concentration-time profiles of the 16 ginsenosides in normal and scopolamine-treated rats following oral administration of RGS at a single dose of 100 mg/kg.

The validated method was successfully applied to pharmacokinetic studies of 16 ginsenosides in rat after the oral administration of RGS extract. The mean plasm concentration-time profiles are shown in Figure 3, and the pharmacokinetic parameters are presented in Table 2.

As shown in Table 2, the pharmacokinetic parameters of Rg1, Rg2, Rh3, Rg5 and Rk1 in SA-treated group had higher AUC, MRT and Cmax values; There was a significant difference in AUC values and Cmax between the SA-treated group and the normal group (*p* < 0.05), which indicates that the bioavailability of Rg1, Rg2, Rh3, Rg5 and Rk1 in the SA-treated group was relatively higher. Moreover, the Tmax values for Rg3 and Rg5 of SA-treated group were higher as well, which indicates that in SA-treated group Rg3 and Rg5 were absorbed and eliminated more slowly. The Cmax values of Rk3, Rh1, Rh2 and Rh4 in SA-treated group were also higher than normal group, but the difference in AUC values between two groups was not significant. There was no significant difference in Tmax, AUC and Cmax value of Rb1, Rb2 Re, Rc, Rd and Rf between SA-treated and normal group. The results indicated that the absorption and concentration of Rg1, Rg2, Rh3, Rg5 and Rk1 were increased due to SA. As the SA-treated group can better absorb the ginsenosides, the host response to SA-treatment may be induced through the nervous system or immune system. This may be due to the difference of absorption and metabolism mechanisms between the different body statuses.

#### 2.2.3. Pattern Recognition Analysis

**Figure 4:**
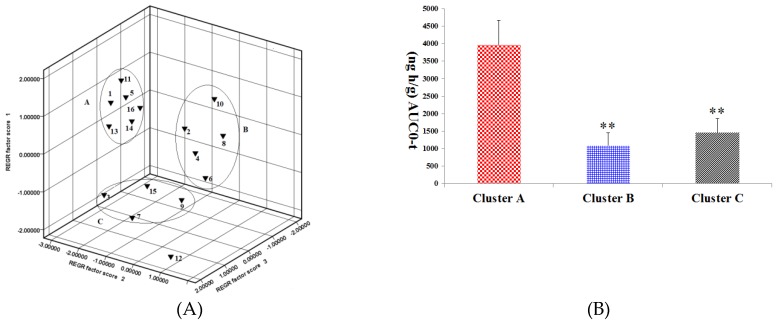
(**A**) Principal component analysis (PCA) scores plot of the 16 ginsenosides absorbed in rat plasma after oral administration of RGS. 1: Rg1, 5: Rg2, 11: Rh4, 13: Rk1, 14: Rg5 and 16: Rh3 were grouped in cluster A; 2: Re, 4: Rb1, 6: Rh1, 8: Rb2 and 10: Rk3 were grouped in cluster B; 3: Rf, 7: Rc, 9: Rd and 15: Rh2 were grouped in cluster C; (**B**) AUC0-t among cluster A, cluster B and cluster C. ** *p* < 0.01, compared with cluster A.

In pharmacokinetic analysis, a pattern recognition analysis (PCA) method was employed to evaluate the pharmacokinetics of the ginsenosides. This method provided reliable and rapid information on the time-course of the ginsenosides absorbed from RGS. The pharmacokinetic parameters were first normalized and then visualized using a pattern recognition method. Based on the PCA score plot of pharmacokinetic data, the 16 ginsenosides were grouped into 3 separate clusters according to difference of kinetic profiles, as shown in Figure 4A. Rg1, Rg2, Rh4, Rk1, Rg5 and Rh3 were grouped in cluster A; Re, Rb1, Rh1, Rb2 and Rk3 were allocated in cluster B; Rf, Rc, Rd and Rh2 were distributed in cluster C.

## 3. Discussion

It has been demonstrated that combined administration of herbal drugs can affect the pharmacokinetics of drugs [26]. To guarantee safety and efficacy of herbs in clinical applications, it is necessary to study the pharmacokinetics of active compounds in herbs in the pathological state. AD is a disease caused by complicated etiological and pathological factors [27,28,29]. Ginseng and ginsenosides have attracted increasing attention owing to their neuroprotective properties. As a key component of ginseng, saponin prevents memory impairment in mice that are subjected to electroconvulsive shocks [30]. Ginsenoside Rg2 improves the recognition deficits in rats induced by cyproheptadine [31]. According to recent studies, Rg1 improves the scopolamine-induced learning ability impairment in rats, while Rb1 and Rd do not [32]. Bioactive components can be absorbed into the tissues or blood and sustain effective concentrations for a long time as well, which is the distinguishing feature for them to be screened out. Table 2 shows pharmacokinetic parameters of the three clusters, in which there is no significant difference in t1/2 and MRT among the three clusters. However, cluster A has Cmax and AUC, which are not owned by clusters B and C (*p* < 0.01). As shown in Figure 4B, the AUC value cluster A is two to seven times larger compared with that of cluster B or C, indicating higher absorption of compounds in cluster A. Rg1 and Rg5 in cluster A showed neuroprotective effects as they enhanced the scavenging and inhibited neuronal apoptosis and the formation of free radicals in the brain [33,34]; It was proved that Rg2 played a role in preventing glutamate-induced neurotoxicity in PC12 cells [35]. In contrast, Rg5 was found to be able to increase CREB activation and BDNF expression and suppress acetylcholinesterase activity so as to exert a neuroprotective effect [1,36]. Based on these results, it could be inferred that Rg2, Rh3, Rh4 and Rk1 in cluster A might have a neuroprotective activity. There was a significant difference in pharmacokinetic parameters of ginsenosides between normal and scopolamine-induced rats. The transformations of ginsenosides in vivo may be a reason that leading to the different pharmacokinetic behaviors of ginsenosides in normal and pathological model rats. For example, Re and Rg1 were decreased slightly, whereas Rg2 and Rh1were increased in the presence of human intestinal microflora [37]. Moreover, many studies have reported that ginsenosides transformed into smaller deglycosylated forms such as Rb1→Rd, F2, Rg3, CK [38], Re, Rb1, Rc→Rg1, Rd, CK [39] and Rb1→Rd, Rg3 [40] by bioconversion methods. This may lead to change in the content of Rg2, Rg3, Rh1 and other rare ginsenosides. However, there is no report about extent and velocity of transformations for these ginsenosides in gastrointestinal tract. Gut–brain axis refers to the bidirectional conduit that communicates the brain with the gut [41]. Numerous findings suggest that the enteric nervous system modulates the immune response and the gut’s function via the gut-brain axis [42]. Scopolamine cause neurotrosis in rats and lead to intestinal dysfunction. Since ginsenosides are mainly passively absorbed by intestinal mucosal membranes [43] the lower absorption and higher elimination in model rats might be due to scopolamine-induced intestinal dysfunction.

According to previous studies, SA-induced amnesia may increase oxidative stress in the brain [5]. However, increased oxidative and nitrosative stresses were found to be associated with elevated MDA in rat brains in this study. SA was also found to disturb the metabolism of glutathione, thereby intensifying the level of lipids peroxidation in the brain [44]. However, through pretreatment of rats with RGS, the increase in MDA in rat brain can be relieved. GSH played a role in the cellular defense system against oxidative damage [45]. The levels of the GSH, CTA and SOD were significantly decreased by injection of scopolamine. However, this situation was effectively suppressed by pretreatment of rats with RGS, which indicated RGS is an ideal type of antioxidant. Acetylcholinesterase inhibitors play a pivotal role in treatment of AD, and can achieve sound curative effects [46]. Relevant studies reported that ginsenosides might also act as an acetylcholinesterase inhibitor [1,36]. Currently, we are focusing on further study to support the observed results.

## 4. Materials and Methods

### 4.1. Chemicals and Materials

**Figure 5:**
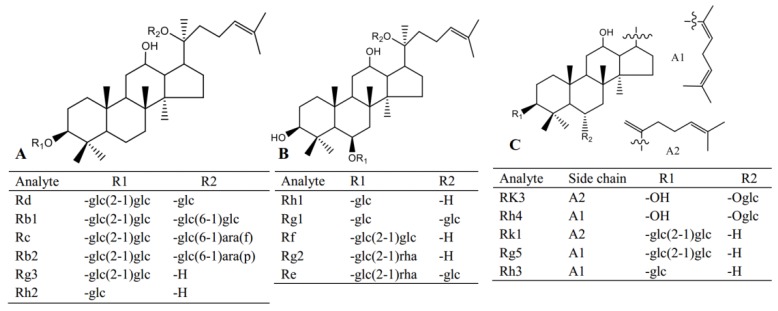
The structures of the analytes in this study.

Red ginseng used in this study was purchased from Fusong, Jiling province; standard compounds including Re, Rg1, Rf, Rb1, Rg2, Rh1, Rb2, Rc, Rd, Rk3, Rh4, Rk1, Rg3, Rg5, Rh2, Rh3, digoxin and scopolamine (Figure 5) were obtained from Yuanye Biological Technology Co., Ltd. (Shanghai, China). HPLC grade methanol and acetonitrile were purchased from Fisher Scientific (Pittsburgh, PA, USA). Malondialdehyde (MDA), glutathione (GSH), superoxide dismutase (SOD) and catalase (CAT) assay kits were purchased from Meibiao Biological Technology Co., Ltd. (Jiangsu, China).

### 4.2. Animals

Male clean Sprague–Dawley rats, with weights varying within 220–230 g were provided by Changchun Yisi Laboratory Animal Technology Co., Ltd. The rats were fed at normal temperature ranging from 22 to 25 °C, under relative humidity of 50–60%, and in the light condition of a 12h light/dark cycle. Moreover, they had free and access to food and water. Before experiment, the rats were fasted for 12 h. Animal experiments were carried out according to the Guidelines of the Care and Use of Laboratory Animals.

### 4.3. Preparation of Red Ginseng Saponins

Red ginseng (1000 g) was sliced and crushed to pieces, and then decocted in water (1000 mL) overnight. After that the obtained solution was filtered, concentrated to 200 mL under reduced pressure at 60 °C, and purified by macroporous resin. Finally, the RGS were dissolved into 100 mL water.

### 4.4. Assessment of Oxidation Stress

#### 4.4.1. Drug Administration

Rats were randomly divided into five groups (n = 6). The control group was injected with saline 10 mL/kg; SA group was injected with scopolamine at a dose of 1 mg/kg for 15 days via intraperitoneal (i.p.) injection; and SA + RGS group was applied with SA + RGS (50, 100, 150 mg/kg, respectively) for 15 days.

#### 4.4.2. Measurements of Biochemical Parameters

After euthanized on day 15, the rat brains rapidly were removed. The samples were weighed and homogenized in 0.05 M sodium phosphate buffer (pH 7.4) with a homogenizer at a speed of 3500 rpm. The obtained supernatants were used for further GSH, SOD, CAT activity assessments as well as MDA by using the corresponding assay kits.

### 4.5. PK Study

#### 4.5.1. Drug Administration

The SD rats were randomly allocated into two groups, i.e., normal group and SA-treated group. SA-treated rats were treated with scopolamine (1 mg/kg, i.p.) 30 min after treatment with SA, the rats were orally administrated with RGS by a single dose of 100 mg/kg.

#### 4.5.2. Preparation of Blood Sample

Blood samples were collected from the postocular vein at 0.083, 0.25, 0.5, 1, 2, 3, 4, 6, 8, 12 and 24 h after SA injection. Blood sample immediately was centrifuged at 18,000 rpm for 10 min. Plasma was withdrawn (0.15 mL) and diluted with 0.3 mL of acetonitrile and 20 µL of the IS (6 µg/mL). After 30 min, the mixture was clarified by centrifugation at 18,000 rpm for 10 min at 4 °C. The supernatant was dried under a stream of N2, and the residue was dissolved in 0.1 mL of methanol and stored at −20 °C for further analysis.

#### 4.5.3. Analytical Condition

The UPLC-MS/MS system (Waters Xeve TQ, Waters, Cork, Ireland) with a column C18 (ACQUITY, 1.7 μm, 2.1 × 50 mm, Waters, Cork, Ireland) were adopted at 35 °C. Water (A) and acetonitrile (B) were used as mobile phases at a flow rate of 0.4 mL/min. The treatment of elution was performed according to following pattern: 10% B for 0–5.0 min; 10→15% B for 5.0–8.0 min; 15→25% B for 8.0–12.0 min; 25→40% for 12.0–20.0 min; 40→50% B for 20.0–25.0 min; 50→65% B for 25.0–30.0 min. With regard to mass detection, the negative mode of electrospray ionization source was adopted. The optimized parameters of multiple reactions monitoring (MRM) system including collision energy, Q1 Pre Bias, Q3 Pre Bias, collision energy and MRM transition of the 16 ginsenosides are shown in Table 1.

### 4.6. Statistics

Based on a non-compartmental method, WinNonlin software (Phoenix, version 6.1) was employed to calculate the pharmacokinetic parameters. The statistical difference between groups was analyzed by One-Way ANOVA analysis using SPSS Version 17.0. (SPSS Inc., Chicago, IL, USA). The difference between groups was regarded significant when *p* < 0.05. We first normalized the pharmacokinetic data and then visualized them based on principal component analysis (PCA).

## 5. Conclusions

After SA injection, the GSH, CAT and SOD levels were significantly decreased. Pretreatment of rats with RGS increased GSH, CAT and SOD levels and significantly decreased MDA levels. SA-treated rats could lead to the changes of pharmacokinetic behaviors of ginsenosides. The 16 ginsenosides were grouped into three separate clusters according to a PCA score plot based on pharmacokinetic data. The results suggested RGS have significant protective effects against scopolamine-treated cerebral injury.

## Figures and Tables

**Table 1:** Optimized precursor/product ion pairs and MRM parameters of the 16 analytes with digoxin (IS) in the negative ion mode.

Analyte	Precursor ion→Product ion	Q1Pre Bias (V)	Collison (V)	Q2 Pre Bias(V)
Rg1	799.5→637.4	23	25	31
Re	945.5→637.4	20	40	32
Rf	799.5→475.4	24	24	32
Rb1	1107.6→945.5	10	39	26
Rg2	783.5→475.4	10	40	33
Rh1	637.4→475.3	19	28	34
Rc	1077.6→945.5	10	45	33
Rb2	1077.6→783.5	32	47	39
Rd	945.6→783.4	15	36	40
RK3	679.4→619.4	10	20	32
Rh4	679.4→619.4	25	30	32
Rg3	783.4→621.3	25	32	33
Rk1	765.5→603.4	23	30	29
Rg5	765.5→603.4	23	30	29
Rh2	621.4→459.3	15	25	32
Rh3	663.5→603.4	12	15	33
Digoxion	799.4→649.3	10	10	31

**Table 2:** Pharmacokinetic parameters of 16 ginsenosides in rat plasma following oral administration of RGS. All data are expressed as mean ± S.D. (n = 5).

Compounds	Ginseng	AUC _0-t_ (ng h/g)	MRT (h)	T_1/2_ (h)	T Max (h)	C Max (ng/g)
Rg1	normal	568.9 ± 10.3 **	15.6 ± 2.1 **	9.6 ± 1.5 **	4.0 ± 1.2 *	20.5 ± 2.5 **
	model	664.5 ± 15.2	16.5 ± 3.2	10.2 ± 1.2	4.0 ± 0.9	32.2 ± 2.1
Re	normal	278.3 ± 9.1 *	9.6 ± 1.2	12.3 ± 2.6 *	0.5 ± 0.4 *	25.6 ± 2.6 *
	model	298.6 ± 21.2	8.6 ± 1.1	11.3 ± 2.9	0.5 ± 0.1	24.9 ± 6.9
Rf	normal	80.2 ± 11.4	6.5 ± 1.2	6.6 ± 1.2 *	2.0 ± 0.6	17.6 ± 5.9
	model	84.5 ± 12.3	7.4 ± 1.3	7.5 ± 1.5	2.0 ± 0.2	18.8 ± 2.6
Rb1	normal	255.6 ± 7.4	6.2 ± 1.8 *	4.3 ± 0.8	4.6 ± 1.1 **	35.2 ± 4.9 *
	model	298.9 ± 11.7	6.5 ± 1.5	4.8 ± 1.1 *	4.1 ± 0.8	34.8 ± 2.7
Rg2	normal	368.1 ± 13.9 **	22.3 ± 1.4 *	19.8 ± 2.5 *	1.5 ± 0.8 *	15.3 ± 5.6 *
	model	456.9 ± 10.2	26.5 ± 3.6	18.9 ± 1.6	1.5 ± 0.6	26.3 ± 4.8
Rh1	normal	215.5 ± 12.1 *	9.6 ± 2.1 *	9.8 ± 2.5 *	1.0 ± 0.5 *	41.2 ± 4.6
	model	278.6 ± 12.8	11.2 ± 2.0	8.9 ± 2.1	1.5 ± 0.3	46.9 ± 7.8
Rc	normal	102.3 ± 20.2 *	9.6 ± 1.5 *	8.9 ± 3.2	1.5 ± 3.2	19.1 ± 2.9 *
	model	109.8 ± 10.2	8.9 ± 2.2	7.9 ± 3.1	1.5 ± 0.5	21.0 ± 4.8
Rb2	normal	89.6 ± 9.3 *	8.6 ± 2.1 **	6.5 ± 1.5	1.5 ± 0.2 *	18.5 ± 2.5 **
	model	90.3 ± 8.2	7.9 ± 1.2	6.9 ± 1.2 *	1.5 ± 0.2	17.2 ± 2.1
Rd	normal	321.5 ± 9.1 *	8.6 ± 1.2	5.5 ± 5.6 *	1.5 ± 0.4 *	47.5 ± 2.6
	model	331.2 ± 11.2	7.7 ± 1.3	6.2 ± 1.9	2.0 ± 0.8	45.6 ± 2.9
Rg3	normal	468.1 ± 12.4 **	24.3 ± 1.2	21.3 ± 2.2 *	1.5 ± 0.6	29.6 ± 5.9 **
	model	556.9 ± 14.3	22.5 ± 2.1	19.8 ± 1.5	4.0 ± 0.6	32.3 ± 4.6
Rk1	normal	556.8 ± 13.4 **	21.3 ± 1.8 *	18.1 ± 9.8	6.0 ± 0.9 **	32.1 ± 5.9 *
	model	736.8 ± 17.7	26.8 ± 2.5	29.3 ± 2.1 *	6.0 ± 0.8	50.1 ± 3.7
Rg5	normal	415.6 ± 20.1 **	22.2 ± 1.4 *	17.1 ± 2.5 *	4.0 ± 1.8 *	34.4 ± 2.6 *
	model	569.8 ± 10.2	25.6 ± 2.6	27.3 ± 3.6	8.0 ± 1.6	37.3 ± 2.8
Rh4	normal	521.3 ± 9.9 **	11.2 ± 2.1 *	8.8 ± 1.5 **	4.0 ± 0.5 *	35.6 ± 3.6
	model	745.6 ± 11.1	12.3 ± 2.0	9.9 ± 2.1	4.0 ± 0.3	38.7 ± 4.8
Rk3	normal	102.3 ± 9.2	9.6 ± 1.5 *	6.5 ± 0.9 *	1.0 ± 0.2	14.2 ± 3.9 *
	model	112.3 ± 8.2	8.9 ± 1.8	6.9 ± 2.1	1.5 ± 0.5	16.6 ± 2.8
Rh2	normal	356.8 ± 12.8	12.3 ± 2.1 *	9.8 ± 2.5	2.0 ± 0.5 *	26.9 ± 2.6 **
	model	369.8 ± 8.0	11.8 ± 2.0	7.6 ± 2.1	2.0 ± 0.3	30.2 ± 4.8
Rh3	normal	468.9 ± 9.2 **	15.6 ± 3.5 *	12.6 ± 3.2 *	14.4 ± 1.2	24.3 ± 3.9 *
	model	798.9 ± 10.2	19.8 ± 2.8	13.6 ± 3.1	10.2 ± 1.5	37.6 ± 4.8

** *p* < 0.01; * *p* < 0.05 compared with AD group.

## Supplementary Materials

The following are available online at https://www.mdpi.com/1420-3049/24/11/2136/s1, Table S1: Regression data and LLOQ of the analytes determined (n = 6), Table S2: Accuracy and precision of the analytes in blank plasma, Table S3: Extraction recovery of the analytes in rat plasma (n = 3), Table S4: The stability of the analytes under different conditions.
