# Supplementary material for: Neuroprotective Effects of Red Ginseng Saponins in Scopolamine-Treated Rats and Activity Screening Based on Pharmacokinetics
Source: Molecules. 2019 Jun 6;24(11):2136. doi: 10.3390/molecules24112136 (PMC6600263; doi:10.3390/molecules24112136)
Supplement: Supplementary file 1 [file molecules-24-02136-s001.pdf]

Validation of LC/MS-MS method

The stock solutions of 16 standards were prepared in methanol. The working solutions of the analytes were prepared by diluting stock solutions with methanol and then blank rat plasma, so that the calibration standards were in the concentrations of 640, 320, 160, 80, 20, 5, 1.25 ng/mL for Rg1, Rf and Rh1; 1280, 640, 320, 160, 40, 10, 2.5 ng/mL for Rb1, Rb2, Rd and Rh3; 128, 64, 32, 16, 4, 1, 0.5 ng/mL for Re and Rg3; 768, 384, 192, 96, 24, 6, 1.5 ng/mL for Rc; 2560, 1280, 640, 320, 80, 20, 5 ng/mL for Rh2; 640, 320, 160, 80, 40, 20, 5 ng/mL for Rh4 and Rk1; 1280, 640, 320, 160, 80, 40, 10 ng/mL for Rg5; 320, 160, 80, 40, 20, 10, 2.5 ng/mL for Rk3; 128, 64, 32, 16, 8, 4, 2 ng/mL for Rg2. The IS solution was prepared and diluted to 1 g/mL with methanol. All solutions obtained were stored at 5 ºC.

Table S1. Regression data and LLOQ of the analytes determined (n = 6).

Composition	Linear Regression Equation	R^2	Linear Range (ng/mL)	LLOQ (ng/mL)
Rg1	Y=0.112x+0.05	0.998	1.25-640	1.0
Re	Y=0.021x+0.22	0.999	1-256	1.0
Rf	Y=0.299x+0.06	0.995	1.25-640	1.0
Rb1	Y=0.114x+0.12	0.999	2.5-1280	1.0
Rg2	Y=0.023x+0.11	0.997	2-128	1.0
Rh1	Y=0.014x-0.12	0.998	1.25-640	1.0
Rc	Y=0.004x+0.02	0.992	1.5-768	1.0
Rb2	Y=0.213x+0.01	0.998	2.5-1280	2.0
Rd	Y=0.121x+0.12	0.993	2.5-1280	2.0
Rg3	Y=0.092x+0.03	0.994	0.5-256	1.5
Rk1	Y=0.014x+0.02	0.992	5-640	2.5
Rg5	Y=0.022x+0.14	0.997	5-1280	1.0
Rh4	Y=0.024x-0.13	0.995	5-640	1.5
Rk3	Y=0.012x+0.03	0.996	2.5-320	2.0
Rh2	Y=0.024x-0.02	0.995	5-2560	1.0
Rh3	Y=0.014x+0.01	0.996	2.5-1280	1.0

Table S2. Accuracy and precision of the analytes in blank plasma.

Com.	Spiked (ng/mL)	Intra-Day (n = 5) Accuracy (%)	Intra-Day (n = 5) Precision RSD%	Inter-Day (n = 5) Accuracy (%)	Inter-Day (n = 5) Precision RSD%
**Rg1**	640	101.1	7.2	102.1	3.5
	80	98.5	4.5	89.6	5.6
	1.25	102.3	5.6	96.5	4.5
Re	128	85.3	4.3	101.3	5.5
	16	89.6	5.8	102.3	4.6
	0.5	91.2	7.6	100.3	3.6
Rf	640	96.2	8.1	85.8	4.7
	80	93.2	6.5	89.3	5.4
	1.25	94.5	4.9	98.6	4.6
Rb1	1280	102.6	5.5	91.2	4.9
	160	101.3	6.9	90.2	7.4
	2.5	98.6	5.8	95.6	5.9
Rg2	128	92.4	4.8	87.5	5.4
	16	98.3	3.2	89.6	4.6
	2	89.3	5.6	89.7	5.6
Rh1	640	95.6	7.5	96.2	2.2
	80	96.3	8.7	99.6	3.5
	1.25	86.4	5.6	98.5	4.6
Rc	768	93.3	4.8	99.5	2.3
	96	96.5	6.5	102.3	5.9
	1.5	98.6	4.5	101.3	5.6
Rb2	1280	95.2	8.4	101.5	6.5
	160	104.3	7.4	100.2	6.1
	2.5	102.3	5.9	99.6	3.5
Rd	1280	83.1	4.8	105.3	5.5
	160	93.2	3.6	89.6	4.9
	2.5	89.4	5.6	88.3	7.1
Rg3	128	99.6	3.7	88.6	3.2
	16	86.3	7.8	94.2	4.6
	0.5	87.6	5.9	99.5	5.8
Rk1	640	86.5	6.9	104.6	4.6
	80	96.3	6.9	102.5	4.5
	5	96.5	7.4	98.5	5.6
Rg5	1280	101.2	5.1	95.7	3.8
	160	103.5	4.2	99.7	2.3
	5	98.5	3.2	98.5	3.8
Rh4	640	92.2	6.2	104.1	7.5
	80	90.9	5.4	102.3	6.5
	5	95.4	3.2	100.4	5.4
Rk3	320	83.2	7.3	82.9	4.6
	40	85.6	5.8	91.2	3.5
	2.5	89.7	7.6	89.6	4.5
Rh2	2560	83.3	5.5	84.3	5.3
	320	79.9	4.6	88.6	5.1
	5	85.3	5.9	87.5	4.1
Rh3	640	98.5	3.2	98.5	5.8
	80	93.2	5.2	104.5	7.5
	2.5	91.9	5.1	101.3	7.8

Table S3. Extraction recovery of the analytes in rat plasma (n = 3).

Com.	Spiked (ug/mL)	Extraction Recovery (%)	RSD (%)
Rg1	640	83.5	8.3
	80	84.2	6.3
	1.25	84.2	8.6
Re	128	83.8	11.7
	16	88.1	7.8
	0.5	81.3	9.1
Rf	640	86.7	6.4
	80	89.4	9.8
	1.25	81.2	7.3
Rb1	1280	83.1	7.2
	160	81.8	9.6
	2.5	84.6	8.8
Rg2	128	83.4	4.5
	16	79.9	8.6
	2	85.5	10.2
Rh1	640	86.3	9.2
	80	79.2	11.4
	1.25	83.5	5.6
Rc	768	87.1	9.3
	96	76.5	8.2
	1.5	84.3	7.4
Rb2	1280	88.5	8.3
	160	84.2	6.3
	2.5	89.2	8.6
Rd	1280	83.7	10.8
	160	89.1	7.8
	2.5	83.5	9.4
Rg3	128	86.7	6.4
	16	89.4	9.1
	0.5	86.2	8.3
RK1	640	83.1	7.3
	80	89.8	7.6
	5	84.6	8.8
Rg5	1280	84.7	9.5
	160	79.9	8.6
	5	85.7	7.2
Rh4	640	86.3	9.2
	80	79.7	10.4
	5	83.5	7.6
Rk3	320	89.1	9.3
	40	76.5	9.2
	2.5	83.6	7.4
Rh2	2560	86.3	10.2
	320	89.1	8.4
	5	87.9	5.6
Rh3	640	87.9	7.3
	80	77.5	9.2
	2.5	88.3	7.9

Table S4. The stability of the analytes under different conditions.

Com.	Spiked (ug/mL)	Accuracy (mean ± S.D.%)			
		Short-Term Stability	Long-Term Stability	Freeze–Thaw Stability	Post-Preparative Stability
Rg1	640	101.0±6.3	102.5±8.1	105.1±3.5	94.6±6.4
	80	92.2±7.1	102.1±9.2	105.0±8.2	105.1±6.2
	1.25	93.1±7.5	93.6±5.6	95.3±4.0	94.4±7.4
Re	128	102.2±9.1	94.6±4.2	95.3±6.5	94.5±7.5
	16	93.4±4.3	98.7±5.2	96.5±7.6	95.5±6.5
	0.25	92.6±4.2	93.2±7.4	99.0±7.6	92.8±6.5
Rf	640	92.3±4.3	94.6±8.7	92.5±6.7	108.8±7.8
	80	95.6±5.2	101.3±9.6	105.6±6.5	96.8±8.8
	1.25	105.3±4.0	101.5±2.7	106.3±5.4	96.1±8.1
Rb1	1280	90.3±5.3	96.6±7.7	96.2±8.4	94.1±4.2
	160	94.5±5.5	107.6±6.8	103.3±6.0	102.2±7.2
	2.5	94.4±7.9	97.9±8.7	94.5±8.4	94.2±8.2
Rg2	128	93.2±2.4	102.9±5.3	104.2±3.4	103.3±4.3
	16	102.4±3.2	93.7±7.7	93.3±9.3	95.6±7.3
	1	95.3±8.7	91.3±7.6	95.3±6.6	97.2±7.9
Rh1	640	102.4±5.1	103.5±8.4	108.5±6.3	102.4±4.4
	80	101.1±6.0	108.1±6.7	103.2±8.1	94.8±7.8
	1.25	105.0±5.1	102.0±8.2	103.7±7.0	95.4±8.8
Rc	768	95.2±4.4	93.2±5.2	92.3±8.3	104.7±7.8
	96	97.6±2.7	105.2±9.4	103.5±5.5	104.4±5.8
	1.5	94.2±4.7	102.8±4.2	100.3±7.3	92.9±3.8
Rb2	1280	101.0±6.3	102.5±8.1	105.1±3.5	94.6±6.4
	160	92.2±7.1	102.1±9.2	105.0±8.2	105.1±6.2
	2.5	93.1±7.5	93.6±5.6	95.3±4.0	94.4±7.4
Rd	1280	102.2±9.1	94.6±4.2	95.3±6.5	94.5±7.5
	160	93.4±4.3	98.7±5.2	96.5±7.6	95.5±6.5
	2.5	92.6±4.2	93.2±7.4	99.0±7.6	92.8±6.5
Rg3	128	92.3±4.3	94.6±8.7	92.5±6.7	108.8±7.8
	16	95.6±5.2	101.3±9.6	105.6±6.5	96.8±8.8
	0.5	105.3±4.0	101.5±2.7	106.3±5.4	96.1±8.1
RK1	640	90.3±5.3	96.6±7.7	96.2±8.4	94.1±4.2
	80	94.5±5.5	107.6±6.8	103.3±6.0	102.2±7.2
	5	94.4±7.9	97.9±8.7	94.5±8.4	94.2±8.2
Rg5	1280	93.2±2.4	102.9±5.3	104.2±3.4	103.3±4.3
	160	102.4±3.2	93.7±7.7	93.3±9.3	95.6±7.3
	5	95.3±8.7	91.3±7.6	95.3±6.6	97.2±7.9
Rh4	640	102.4±5.1	103.5±8.4	108.5±6.3	102.4±4.4
	80	101.1±6.0	108.1±6.7	103.2±8.1	94.8±7.8
	5	105.0±5.1	102.0±8.2	103.7±7.0	95.4±8.8
Rk3	320	95.2±4.4	93.2±5.2	92.3±8.3	104.7±7.8
	40	97.6±2.7	105.2±9.4	103.5±5.5	104.4±5.8
	2.5	94.2±4.7	102.8±4.2	100.3±7.3	92.9±3.8
Rh2	2560	102.4±5.1	103.5±8.4	108.5±6.3	102.4±4.4
	320	101.1±6.0	108.1±6.7	103.2±8.1	94.8±7.8
	5	105.0±5.1	102.0±8.2	103.7±7.0	95.4±8.8
Rh3	640	95.2±4.4	93.2±5.2	92.3±8.3	104.7±7.8
	80	97.6±2.7	105.2±9.4	103.5±5.5	104.4±5.8
	2.5	94.2±4.7	102.8±4.2	100.3±7.3	92.9±3.8
